# Impact of Severity of Sickle Cell Anemia on Auditory Discrimination Ability and Speech Perception in Noise

**DOI:** 10.1055/s-0044-1789255

**Published:** 2025-01-10

**Authors:** Preeti Sahu, Animesh Barman

**Affiliations:** 1 Department of ENT & HNS, All India Institute of Medical Sciences, Raipur, Raipur, Chhattisgarh, India; 2 Department of Audiology, All India Institute of Speech and Hearing, Mysore, Karnataka, India

**Keywords:** sickle cell anemia (SCA), speech perception, auditory function

## Abstract

**Introduction** Sickle cell anemia (SCA) is a genetic disorder with clinical manifestations due to circulatory changes, leading to adverse effects on the auditory system that might impact auditory processing, such as auditory discrimination and speech perception ability. This condition is associated with the severity level of anemia.

**Objective** The purpose of the present study was to investigate the influence of anemia severity on auditory discrimination ability and speech perception in noise among SCA patients with normal hearing sensitivity.

**Methods** A total of 52 normal-hearing adults diagnosed with SCA in the age range of 15 to 40 were grouped into mild, moderate, and severe, based on anemia severity. Auditory discrimination tests for frequency, intensity, and duration were evaluated at 500 and 4,000 Hz along with speech perception in noise (SPIN) at 0 dB SNR using the mlp toolbox in the MATLAB software, version 2014a (MathWorks, Natick, MA, USA). The IBM Statistical Package for Social Sciences (SPSS) version 26.0 (IBM Corp., Armonk, NY, USA) was used for statistical analysis.

**Results** The results revealed an increase in median and interquartile range among anemia groups with increasing severity. Additionally, the median scores were found to be poorer for the higher frequency in all auditory discrimination tests than for the lower one. A regression in performance with an increase in severity for the SPIN test was observed.

**Conclusion** The severity of anemia plays an important role in functional auditory processing deterioration. Circulatory changes secondary to SCA affected auditory discrimination processing and speech perception in noise. However, all auditory discrimination abilities are not necessarily affected equally.

## Introduction

A genetic condition known as sickle cell anemia (SCA) is characterized by aberrant red blood cells that can lead to a variety of health issues. 1 Acute pain episodes and multiple chronic organ damage, including to the auditory system, 2 are consequences of this condition along with anemia. Due to circulatory changes, the pathophysiology of SCA culminates in a range of clinical manifestations. 3 As an end-organ system, the auditory system is especially susceptible to vaso-occlusive events. An SCA crisis causes stasis of the labyrinthine artery supplying the inner ear. The organ of Corti and the stria vascularis in the cochlea are impacted by this hypoxia, which eventually results in the loss of outer hair cells—essential for amplifying sound. This is followed by hearing loss and irreversible inner ear damage. 4 Sharp and Orchik 5 and Armstrong et al. 6 have reported altered auditory processing abilities, such as auditory closure, which may be due to these subtle changes in the inner ear combined with neurological complications. 7 Auditory processing includes the ability to understand and interpret information through auditory discrimination, auditory pattern recognition, temporal elements of hearing, and auditory performance in competing or degraded signals. 8

The general capacity to distinguish between two acoustic events is known as auditory discrimination. 9 While duration discrimination skills enable the perception of differences between, for example, “ditch” and “dish” based on the length of the final consonant, frequency discrimination can aid in the perception of sounds that differ in pitch. Lastly, intensity discrimination is considered critical to understanding how speech sounds vary in energy during a conversation or while listening to music, even in infants. 10 Sufficient auditory processing capacity is necessary to perceive speech correctly during a conversation. Both higher-level mechanisms, like cognitive processes, and more general, low-level, systems are dependent on auditory processing in compromised states, like those caused by noise. 11 Any underlying medical condition, like SCA, can lead to physiological changes in the central and peripheral auditory systems, which can impair an individual's ability to perceive speech and directly impact a variety of auditory processing skills. 12

The degree of anemia that each person experiences is one of the main characteristics of SCA. Hemoglobin is a crucial part of red blood cells that carry oxygen; its reduced concentration is referred to as anemia and lower levels indicate a more severe form of the disease. The severity of anemia in SCA can range from mild to severe. The criterion for anemia severity level is shown in Table 1 . 13

**Table 1:** The WHO criteria for anemia and grade of severity based on hemoglobin level

SL no.	Population	Nonanemia (Gm/dL)	Anemia (Gm/dL)		
			Mild	Moderate	Severe
1.	Non-pregnant women (15-years-old and older)	12	11.0–11.9	8.0–10.9	< 8.0
2.	Pregnant women	11	10.0–10.9	7.0–9.9	< 7.0
3.	Men (15-years-old and older)	13	11.0–12.9	8.0–10.9	< 8.0

**Abbreviations:** WHO, World Health Organization; SL, severity level.

The majority of earlier research examined how anemia affected auditory acuity and hearing function. 14 Few studies have reported on the common and critical role hemoglobin concentration may play in the decline of hearing function. An evaluation of auditory discrimination in relation to the severity of anemia, in conjunction with other tests such as speech perception in noise (SPIN), may offer a more comprehensive understanding of the detrimental impact of anemia on subtle anatomical alterations in the auditory system, affecting auditory processing. This includes auditory discrimination and speech perception ability under unfavorable conditions.

## Aims and Objectives

In SCA patients with normal hearing sensitivity, the current study intends to examine the impact of anemia severity on auditory discrimination ability and speech perception in noisy environments.

## Methods

To accomplish the study's goal, a nonexperimental standard group comparison method 15 was used, with a between-subjects design. The study employed nonrandom purposive sampling to select participants from a comparable community and urban area. A total of 52 adults with normal hearing who had been diagnosed with SCA and were between the ages of 15 and 40 participated in the study (of whom 27 were male and 25 were female). Based on the severity of their anemia—all participants were divided into three groups (i.e., mild, moderate, and severe) according to the blood hemoglobin (Hb)S concentration ( Table 2 ). For a minimum of 1 year, participants were enrolled for follow-up in secondary and tertiary health care centers for their conditions.

**Table 2:** Anemia severity, Hb concentration, and demographic details among SCA participants

Anemia Severity	Hb concentration			Gender			Age	
	(gm/dL)	Mean	SD	Male	Female	Total	Mean	SD
Mild	11.0–11.9 (female) 11.0–12.9 (male)	11.57	0.29	10	3	13	25.69	7.80
Moderate	8.0–10.9	9.34	0.91	10	12	22	26.09	6.80
Severe	<8.0	7.29	0.72	7	10	17	26.76	7.73

**Abbreviations:** Hb, hemoglobin; SD, standard deviation.

Every participant underwent an A-type tympanogram, with their hearing thresholds less than or equal to 15 dB HL at frequencies spanning from 250 to 8000 Hz. Speech audiometry revealed a minimum of 80% bilateral speech identification scores (SIS) and a strong correlation between pure tone audiometry (PTA) and speech recognition thresholds (SRT) within 10 dBHL. The distortion product otoacoustic emission (DPOAE) screening revealed that both ears passed. Additionally, it was determined through a structured interview that there was no medical history of neurologic or otologic conditions.

The participation of all the study subjects was entirely voluntary, without monetary compensation. The institute's ethics committee gave approval (reference no. of the project: DOR.9.1/Ph.D/PS25/2022–23) before the experiment started, as per the standard operating procedure. Every participant gave written consent to join the study voluntarily. There were two stages to the study. Phase I was comprised of participant selection and assignment to groups according to the inclusion and exclusion criteria. Phase II involved the investigation of those chosen for all test categories, including the SPIN test and the auditory discrimination tests.

### Auditory Discrimination Tests

Testing for frequency, intensity, and duration was done through an auditory discrimination task. Using the maximal likelihood procedure (mlp) toolbox, which is implemented as mlp in the MATLAB (MathWorks, Natick, MA, USA) software, all auditory discrimination tests were conducted. 16

The stimulus to be presented at the following trial was determined by the threshold set by the previously established psychometric function yielding the highest probability. The maximum likelihood method often converges on a reasonably stable approximation of the psychometric function after approximately 12 trials, which is subsequently used to estimate the threshold. 17 The stimuli were generated at a 44,100 Hz sampling rate. An alternate forced-choice method with three intervals and mlp was used to track a 79.4% correct response criterion.

Every auditory discrimination test was administered according to the previously mentioned protocol, and stimulus presentation and response acquisition were controlled using the mlp toolbox. For every test, there were five to six practice questions available before the actual exam began. All psychoacoustical tests used a binaural test stimulus presented at 85 dB SPL. The 500 and 4,000 Hz test frequencies were used. An Intel Core i5, HP laptop (HP Inc., Palo Alto, CA, USA) with Sennheiser HD-449 headphones (Sennheiser electronic GmbH & Co. KG, Wedemark, Germany) connected provided the stimuli for every test. The headphones' output was adjusted to generate 85 dB SPL for a pure tone at 1,000 Hz in a 6cc coupler.

Difference limen for frequency (DLF) and for intensity (DLI) for pure tones were measured for 500 and 4,000 Hz. 18 The duration discrimination threshold (DDT) was measured for 500 and 4,000Hz. 19 The duration of the anchor tone was 250 milliseconds. 20

### Speech Perception in Noise

**Fig. 1:**
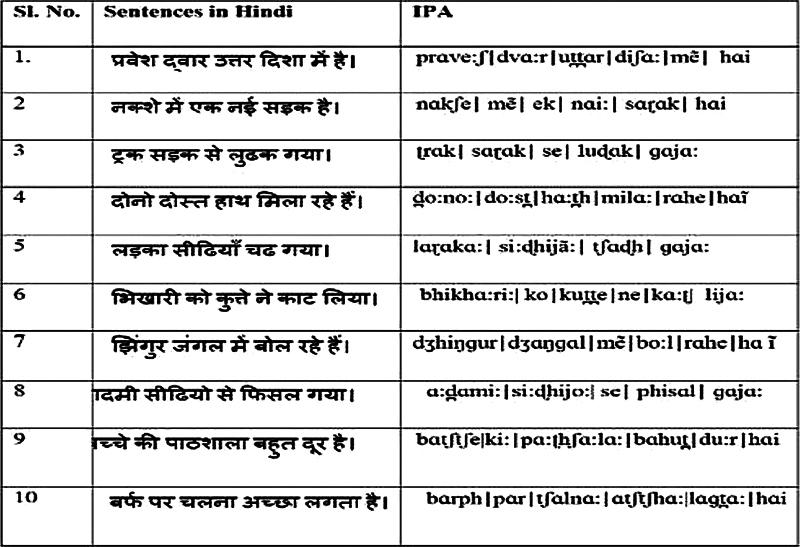
Hindi Sentence list-1 and IPA format created and standardized by Jain et al. 21 utilized for SPIN testing at 0 dB SNR.

Speech perception in noise: To assess speech perception in noise, the Hindi Sentence Test for Speech Recognition in Noise 21 at 0 dB signal-to-noise ratio (SNR) was utilized. To reach this value, each sentence was digitally combined with speech spectrum-shaped noise using MATLAB. Since the sentence list was initially standardized at this level, the stimuli mixed with the noise were presented binaurally using standard Sennheiser-HD200A headphones as transducers at an intensity of 70 dB SPL. 22 The Hindi sentence test List 1 contains a total of 40 keywords ( Fig. 1 ). The participants were instructed to either write down or speak the target sentences out loud. The number of accurate keywords identified at 0 dB SNR was tallied.

## Results

The study's data were statistically analyzed using the IBM SPSS Statistics for Windows (IBM Corp., Armonk, NY, USA), version 26.0. Descriptive statistics were used to estimate each parameter's mean and standard deviation. When the data were subjected to a Shapiro-Wilk test for normality, it was discovered that, for the 3 auditory discrimination tests (DLF, DLI, and DDT) between the frequencies (500–4,000 Hz) and for SPIN, the data did not follow a normal distribution in any of the three anemia groups.

Since the data did not follow a normal distribution, the groups were compared using the median values of all parameters. Table 3 shows the median and inter-quartile range for DLF, DLI, DDT, and SPIN at 0 dB SNR. It can be observed that for all auditory discrimination tests (DLF, DLI, and DDT) at 500 Hz and 4,000 Hz, there is a trend of increase in the median and the interquartile range (IQR) from mild to severe group. For the SPIN test, a regression in performance was noted as the test's severity increased. Furthermore, it was discovered that the median scores for all auditory discrimination tests were higher at 4,000 Hz than at 500 Hz.

**Table 3:** Median and IQR for auditory discrimination tests at 500 and 4,000 Hz, and SPIN across the anemia groups

Anemia severity group		Mild		Moderate		Severe	
Test parameters	Frequencies	Median	IQR	Median	IQR	Median	IQR
DLF	500 Hz	24.52	39.03	51.67	59.11	54.21	56.12
	4,000 Hz	189.52	107.71	97.69	119.15	183.34	60.62
DLI	500 Hz	2.20	1.30	2.50	1.24	3.30	2.50
	4,000 Hz	2.50	1.85	3.20	1.77	5.70	5.35
DDT	500 Hz	68.80	58.88	78.13	36.89	138.05	91.10
	4,000 Hz	83.86	52.51	89.74	52.23	116.22	114.29
SPIN	0 dB SNR	35.00	2.00	33.5	6.00	31.00	6.00

**Abbreviations:** DDT, duration discrimination threshold; DLF, difference limen for frequency; DLI, difference limen for intensity; Hb, hemoglobin; IQR, interquartile range; SNR, signal-to-noise ratio; SPIN, speech perception in noise.

**Fig. 2:**
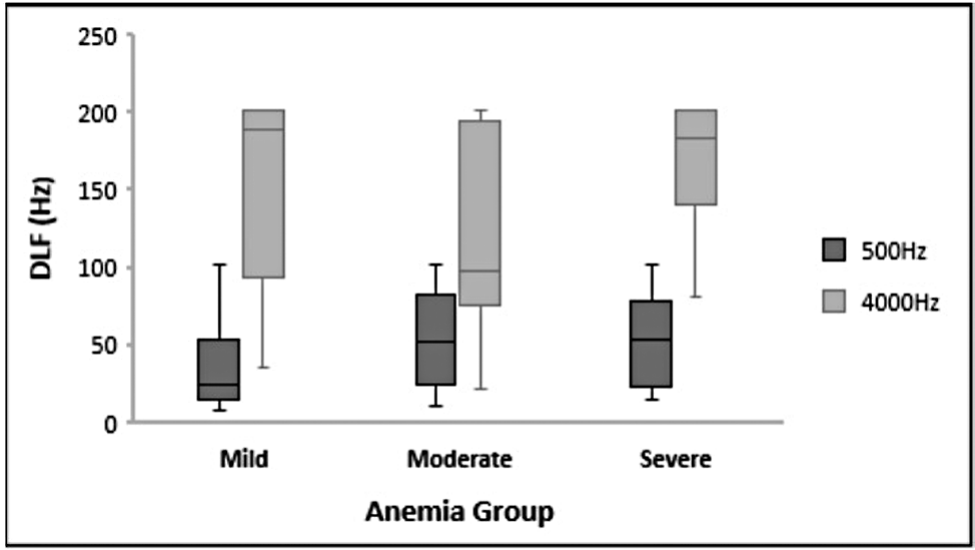
Median and IQR bar for DLF at 500 and 4,000 Hz frequency across the anemia group. No significant differences were obtained between any anemia group for both the test frequencies.

**Fig. 3:**
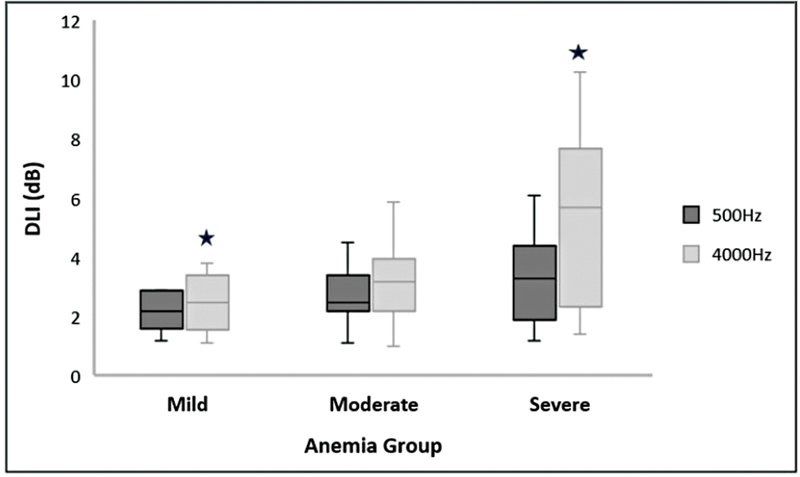
Median and IQR bar for DLI at 500 and 4,000 Hz frequency across the anemia group. Significant differences were obtained between mild and severe anemia groups at a *p* -value < 0.05 for test frequency 4,000 Hz, depicted with the star (  ) marking.

**Fig. 4:**
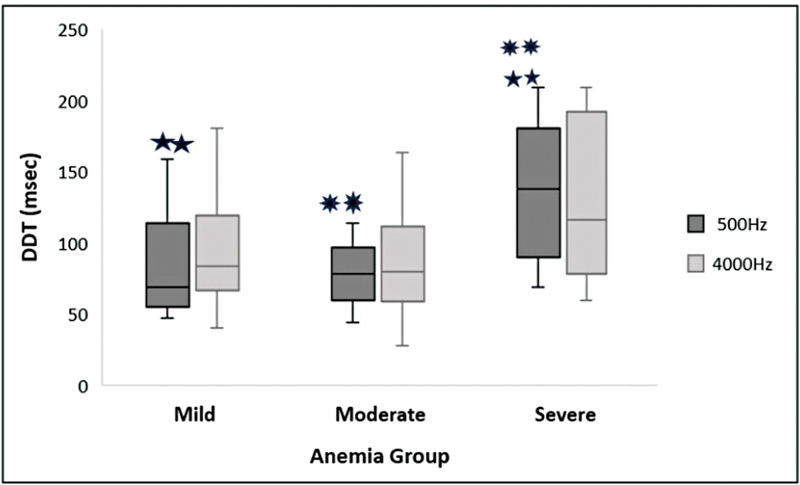
Median and IQR bar for DDT at 500 and 4000Hz frequency across the anemia group. Significant differences were obtained between mild and severe and moderate and severe anemia groups at a *p* -value of < 0.01 for test frequency 500 Hz, depicted a different star(   ) marking.

**Fig. 5:**
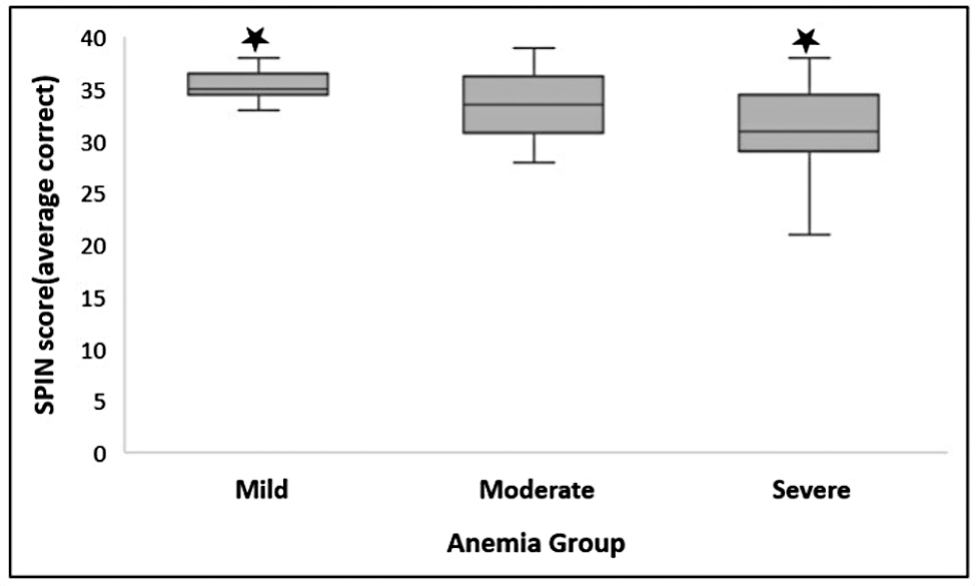
Median and IQR bar for SPIN at 0 dB SNR in the anemia group. Significant differences were obtained between mild and severe anemia groups at a *p* -value of < 0.01, depicted with the star (  ) marking.

Additionally, the significance level of difference for all auditory test results (DLF, DLI, and DDT) at each stimulus frequency (500 & 4,000 Hz) and SPIN at 0 dBHL among the anemia groups was assessed using the Kruskal-Wallis's test. No statistically significant differences were found for DLF (DLF, 500 Hz, χ2(2) = 2.763, *p* = 0.251; 4,000 Hz, χ2(2) = 5.899, *p* = 0.052), Fig. 2 , among the anemia groups. On the other hand, the findings also showed significant differences in DDT at 500 Hz, DLI at 4,000 Hz, and SPIN scores at 0 dB SNR between anemia severity groups. First, DLI's results at 500 Hz are: χ2(2) = 6.989, *p* = 0.371; and 4,000 Hz, χ2(2) = 6.989, *p* = 0.030; as shown in Fig. 3 . As for the DDT, at 500 Hz: χ2(2) = 12.628, *p* = 0.002; and at 4,000 Hz: χ2(2) =12.628, *p* = 0.057, as shown in Fig. 4 . Finally, the SPIN results are: χ2(2) = 9.350, *p* = 0.009, Fig. 5 .

Also, pairwise comparisons of the test parameters with significant variations in Kruskal-Wallis were conducted between the anemia groups. The outcome showed that while there was no significant difference between the mild and moderate groups for any of these three test parameters (DLF, DLI, and DDT), there was a significant difference between the mild and severe anemic groups for all three of them. Furthermore, only DDT at 500 Hz demonstrated significant differences in the group with moderate and severe anemia; DLI at 4,000 Hz, and SPIN at 0 dB SNR did not show any significant differences.

The relationship between the severity of anemia, SPIN, and the results of the auditory discrimination tests (DDT: 500, Hz and DLI: 4,000 Hz) was evaluated using the Spearman correlation. The results showed a significant positive weak correlation between the anemia group and DLI test scores and a positive moderate correlation between the anemia group and DDT test scores indicating the increase in anemia severity leads to higher/poorer scores in individuals (DLI: 4,000 Hz, ρ = 0.364, *p* = 0.008; DDT: 4,000 Hz, ρ = 0.0.430, *p* = 0.001). A moderate negative significant correlation was observed between SPIN and anemia severity revealing poorer/lower scores with an increase in anemic severity (SPIN at 0 dB SNR, 4,000 Hz, ρ = -0.428, *p* = 0.002). Moreover, there was no correlation found between the auditory discrimination test results (DLI: 4,000, Hz and DDT: 500 Hz) and SPIN scores.

## Discussion

The results of all three auditory discrimination tests—DLF, DLI, and DDT—showed that the severity of anemia had a negative impact when the stimulus frequency was increased from 500 to 4,000 Hz. This could have been the result of subtle anatomical changes in the inner ear caused by the stasis of the labyrinthine artery and decreased nutrient and oxygen fulfillment. This further resulted in hypoxia of the Organ of Corti and Stria Vascularis, as well as the death of sensory cells, or the outer and inner hair cells, as a consequence of the pathophysiology of SCA, which is caused by vaso-occlusion and hemolysis. 22 23 Additionally, the lack of normal functioning Hb deficiency and destruction of RBC, the inflammatory mechanism specific to SCA leads to cochlear micromechanics prematurely along with endothelial dysfunction which corrupts endolymphatic K ^+^ recycling causing an imbalance of endo-cochlear potential allowing toxic substances to enter the cochlea seen in the SCA population. 24 These factors, in isolation or combination, might have led to anatomical and functional changes, which further worsened with an increase in the severity of anemia and are responsible for poorer auditory perception in SCA individuals.

Overall auditory discrimination abilities were found to be more affected in higher test frequencies, being 4,000 Hz in the present study, when compared with lower test frequencies, 500 Hz in this case, which may be due to the asymmetrical anatomical distribution of blood supply throughout the length of the cochlea found to be fewer and narrower in the basal high-frequency region at and around 4,000 Hz 25 further deteriorated the functional ability at 4,000 Hz among SCA individuals.

Moreover, on frequency-specific comparisons within each auditory discrimination ability, DDT at 500 Hz and DLI at 4,000 Hz were found to have a statistically significant difference among the anemia groups in the present study, whereas DLF results showed no significant difference between two test frequencies among anemia groups.

The temporal coding is reported to be more important at lower frequencies, which needs the temporal fine structure's information processing. 26 Due to vaso-occlusion throughout the cochlea, even a slight alteration in a low-frequency partition may alter the temporal processing reflected in DDT ability.

However, the excitation pattern at the basilar membrane is limited to the basal region for HF, and poor neural excitation is a consequence of sensory cell insult at the inner ear since it receives fewer circulatory terminals and more necrotic changes secondary to vaso-occlusion and hemolytic process in SCA may result in poor intensity discrimination.

Speech perception at 0 dB SNR was affected significantly by the severity of anemia in individuals with SCA. As for SPIN, it requires intact frequency, intensity, and temporal resolution ability. Though there is no significant difference in DLF in the present study, still there is a trend to a higher value with an increase in severity. Second, the DLI and DDT were found to be affected in study participants. Hence, these factors in cumulation might lead to poorer SPIN findings in SCA individuals.

Auditory discrimination test scores and anemia severity have a significant positive correlation suggesting the use of this measure to evaluate the degree of deterioration in auditory discrimination abilities with anemia increase in SCA individuals. A significant negative low correlation with SPIN results reflects lower scores, which is suggestive of the increasing difficulty of speech perception in the presence of background noise with increasing anemia severity, and a possible measure to use in the clinical condition to evaluate the impact of anemia severity on day-to-day speech perception and/or communication ability in the SCA population.

## Clinical Implications

The results found in this study:

- Raise the need for early identification and intervention in special test populations like SCA.
- Suggest the possible place of insult is in the cochlea, due to SCA.
- Induce the need for inclusion of different auditory processing tests, such as the discrimination ability of frequency, intensity, and duration, in day-to-day clinical practice.
- Help in understanding and choosing appropriate therapeutic and intervention approaches to improve the quality of life of SCA individuals and better counseling.

## Limitations and Future Direction

Although the present study aimed to evaluate the auditory discrimination and speech perception in noise for individuals with SCA of increasing severity level, all the participants were on medication since the time of diagnosis as per the symptoms. This might have led to overall better auditory discrimination and SPIN scores in these participants, even with the increasing severity of anemia in the present study. Also, the length of medication intake might have a direct or indirect influence on the study outcomes that must be studied separately. Since SCA is a fatal disorder, it is difficult to temporarily stop or delay medicine intake in this population.

The impact of auditory processing ability probably can be accurately evaluated through a longitudinal research design for a finer and better understanding of the consequences of anemia severity with preexisting SCA conditions. The comparison of this population with healthy ones would also highlight the actual deviation of auditory processing ability in this population in comparison to non-SCA healthy individuals. Furthermore, it would be helpful to identify the signs of emergence for early intervention, to overcome communication challenges in SCA patients with increasing anemia severity.

## Conclusion

The severity of anemia plays an important role in functional auditory processing deterioration. The present study reflected the correlation between increased impaired auditory discrimination ability and the increase in anemia severity. Circulatory changes secondary to the SCA were probable reasons to affect auditory discrimination processing and speech perception in noise. However, not all auditory discrimination abilities can necessarily be affected equally.
